# Molecular and Genetic Insights into Thoracic Aortic Dilation in Conotruncal Heart Defects

**DOI:** 10.3389/fcvm.2016.00018

**Published:** 2016-06-07

**Authors:** W. Aaron Kay

**Affiliations:** ^1^Department of Medicine, Krannert Institute of Cardiology, Indiana University School of Medicine, Indianapolis, IN, USA

**Keywords:** conotruncal, tetralogy of Fallot, transposition of great arteries, aortic aneurysm, aortic dissection, truncus arteriosus

## Abstract

Thoracic aortic dilation (AD) has commonly been described in conotruncal defects (CTDs), such as tetralogy of Fallot, double outlet right ventricle and transposition of the great arteries, and truncus arteriosus. Several theories for this have been devised, but fairly recent data indicate that there is likely an underlying histologic abnormality, similar to that seen in Marfan and other connective tissue disease. The majority of aortic dissection in the general population occurs after the age of 45 years, and there have been very few case reports of aortic dissection in CTD. Given advances in cardiac surgery and increasing survival over the past several decades, there has been rising concern that, as patients who have survived surgical correction of these defects age, there may be increased morbidity and mortality due to aortic dissection and aortic regurgitation. This review discusses the most recent developments in research into AD in CTD, including associated genetic mutations.

## Introduction

Conotruncal cardiac defects (CTDs) include a variety of congenital heart defects, such as tetralogy of Fallot (TOF), truncus arteriosus (TA), double outlet right ventricle (DORV), and transposition of the great arteries (TGA). These defects represent 5–10% of congenital heart disease and, generally, lead to severe cyanosis, necessitating repair in the newborn period or early in infancy. A common observation in CTD is thoracic aortic dilation (AD). It has been known for over half a century that AD is common in TOF (1, 2). Landmark work by Niwa demonstrated that the incidence of AD in adults with repaired tetralogy approaches 15% (3). Progressive dilation of the neo-aortic root is out of proportion to somatic growth in TGA after arterial switch surgery (4), and AD is found in the majority of patients with TA who survive initial repair (5).

Aortic dilation can become more clinically relevant if it leads to significant aortic valve regurgitation, aortic dissection, or worsened LV–aorta interaction (6). AD can lead to significant morbidity and mortality, with the chief worry being ascending aortic or aortic root dissection, which is often fatal without emergency surgery, and as a result, clinicians seek to evaluate patients to avoid this complication, generally by assessing aortic size on non-invasive imaging, and intervening with elective surgery in a more controlled setting. Specific guidelines exist for elective surgery to prevent dissection in Marfan syndrome and certain other connective tissue disease, but specific guidelines for elective surgery for AD in CTD have not been established (7). One interesting article over several years evaluated the outcomes of 81 adults with CTD and AD who had surgery over several decades and came to the conclusion that elective surgery in CTD should be delayed unless the maximum aortic dimension is at least 5.5 cm, unless there is documented rapid growth of the ascending aorta, or a worrisome family history of aortic dissection or aneurysm (8).

In the past, it was thought that the risk of aortic dissection in these patients was low, perhaps due to the low incidence of systemic hypertension, smoking, and other traditional vascular risk factors in this population (9). However, there have now been four case reports of dissection in TOF (10–13). All these cases except the most recent one dissected at diameters >6 cm. Perhaps, aortic cannulation during the initial repair could be blamed for a dissection late after repair, but this seems unlikely given that the dissections occurred anywhere from 9 years to more than 30 years after the initial repair (10–13). It would stand to reason that, if direct injury to the aorta during initial cannulation was the culprit, there would be a significant number of dissections reported in childhood in the literature, when, in fact, the youngest patient identified in the literature was already 18 years of age, more than 17 years after initial compete TOF repair (12).

Regarding TGA, fortunately, no case reports of aortic dissection in TGA after arterial switch have been published, but perhaps none have occurred due to the relative young age of this population, given that most survivors of the arterial switch procedure are <30 years old. To date, there has only been one case report of an aortic dissection in TGA after Mustard repair (14). In this case, the patient had been lost to cardiology follow-up for over two decades, had several pregnancies, and smoked cigarettes, and it was unknown what size the aorta was prior to dissection. Isolated cases of dissection in CTD have also been found in review of administrative databases (15). There has been only one case report of elective aortic root replacement in a TGA patient after Mustard procedure – which was performed in a 30-year-old man, for an aortic aneurysm, measuring 4.5 cm (16). Still, the majority of patients with dissection in CTD have been older than 45 years of age. However, as the population of CTD survivors’ ages, more patients might be at risk for aortic dissection or other complication.

It is important to note that aortic dissection is not the only concern or cause of morbidity due to AD in patients with CTD. The dilation itself can lead to significant aortic valve regurgitation, which can lead to an increased pulsatile load on the left ventricle (or systemic right ventricle), leading directly to decreased cardiac output, or indirectly *via* decreasing coronary arterial blood flow (17, 18). Aortic regurgitation may additionally worsen not just due to dilation but also due to stiffness of the aortic root (19). The need for aortic valve replacement is fortunately fairly uncommon in CTD, although the presence of aortic regurgitation is fairly common in TGA after arterial switch (3, 20). After arterial switch surgery, Losay and others noted that freedom from aortic regurgitation was 78% at 10 years and 69% at 15 years; however, freedom from aortic valve replacement was 98% at 10 years and 97% at 15 years (21). In a study by Marino and others, severe neo-aortic valve regurgitation was present in 3.7% and trivial to mild regurgitation in 81% of patients at mid-term follow-up (22).

## Possible Mechanisms for Aortic Root Dilation in Conotruncal Defects

There are a few hypotheses for why AD in CTD occurs, independent of standard risk factors, such as hypertension, aging, pregnancy, and smoking. The first is that AD occurs due to hemodynamic stress on the aorta from a chronic right-left-shunt. Evidence to support this hypothesis include data showing that AD is worse with worsened degrees of right ventricular outflow tract stenosis and is worse in patients with pulmonary atresia than in patients with pulmonary stenosis (23). A second hypothesis is that volume loading of the aorta, *via* a surgical systemic-to-pulmonary shunt, will increase flow through the aortic valve, thus leading to dilation of the proximal aorta *via* increased wall stress (24). A longer duration between shunting and complete repair has been found to correlate with AD in repaired TOF (23, 25). One study showed a 12% increase in mean aortic diameter after surgical aortopulmonary shunting (25). Other observations that have been associated with larger aortic dimensions in TOF have included a right rather than left aortic arch and male sex.

Newer data suggest that CTD may be associated with a primary problem with aortic histology, i.e., a true aortopathy. Evidence of aortopathy in TOF has been found early in life, on fetal echocardiography (26), and also on histologic studies. Even in infants, higher histologic grading scores in TOF patients have been seen, thus making it likely that there is an intrinsic abnormality of the aortic tissue leading to dilation, rather than long-term hemodynamic stress (20). Histologic abnormalities were reported in a cohort of 15 repaired TOF patients with ascending aortic aneurysms, and this was further supported in another study demonstrating elastin fragmentation in the ascending aorta in 74.5% of 98 consecutive patients undergoing complete TOF repair (27, 28).

There are many variables that affect the size of the aortic root and ascending aorta in general. Although there have been similarities of aortic root histology seen between Marfan syndrome and some CTDs, it is notable that the risk of dissection in Marfan is significantly higher, which begs the question why the Marfan phenotype is so much more dangerous. It is possible that the histologic abnormality in the aorta found in TOF may be less severe than the abnormality found in Marfan syndrome (27).

Most research regarding AD in CTD predominantly focused on TOF with fewer papers focused on TGA or truncus. DORV is rarely considered by itself in the literature, as it is a diagnosis encompassing a broad spectrum of pathophysiology, depending on where the ventricular septal defect is in an individual patient and also on the relationship of the great arteries to one another. It most commonly presents with TOF-like physiology, wherein the patient has a subaortic ventricular septal defect with pulmonary stenosis. In most of the literature, DORV with this physiology is included as a TOF variant.

Conclusions have been difficult to draw, given differing definitions of what constitutes an aortic aneurysm. Most pediatric centers have used *Z*-scores, whereas most adult studies have looked at either absolute dimensions or dimensions indexed to either body surface area or height. A common definition for “aneurysm” is an observed to expected ratio of >1.5 of the normal population at a specific aortic segment. A large study using cardiac MRI evaluated normal values in a control population and can be used as a helpful baseline (29). Very few studies have performed longitudinal measurements to demonstrate if the AD found in CTD is likely to progress over time. In one study of children with both TOF and TGA, independent predictors of a longitudinal increase in *Z*-scores of the ascending aorta included male sex and presence of aortic regurgitation (30). A study of adults with repaired tetralogy, utilizing MRI as the imaging technique, showed minimal growth in TOF over the course of 3 years, with a very small number of aortas increasing in size from below to above a threshold value of 5 cm (31).

As survival of CTD patients has improved, numerous patients with CTD have been able to have pregnancies of their own. Pregnancy is known to be an independent variable for the structural change of the aortic media, but it is unknown how likely these changes are to regress after delivery and whether the changes that occur with gestation are additive to the normal aging process in this population (27).

## Genetic and Molecular Findings

The investigation of AD in CTD from a molecular and genetic standpoint, compared to the robust database found in Marfan syndrome, is still in its infancy. There have been very few articles investigating the genetic or histologic associations of AD and CTD, to date. Marfan syndrome, with a prevalence of 1:5000, is in most cases due to a mutation in the fibrillin-1 gene (*FBN1*) (32). Fibrillin-1, together with other proteins of the extracellular matrix, forms thread-like microfibrils, which create structural support and elasticity to tissues. Mutations in *FBN1* lead to breakdown of microfibril architecture, which can lead to aortic aneurysms and other complications. There are numerous mutations in the *FBN1* mutation database, with nearly 3000 reported mutations to date, varying from point mutations to large rearrangements.

Research demonstrating that the histology of the aorta in patients with congenital heart disease is similar to that of Marfan patients (27) has led to small studies investigating the role of fibrillin in CTD. Given the much lower incidence of dissection in TOF, it is possible that the histologic abnormality in the aorta found in TOF may be less severe than the abnormality found in Marfan syndrome (27). In a study of 74 consecutive patients undergoing intracardiac repair or TOF, full-thickness aortic wall biopsies were performed, and there was a 50.9% prevalence of *FBN1* gene polymorphisms or mutations in those with a dilated aorta (28). Additionally, the risk of AD was found to be eight times higher in patients with these variants. Abnormal histology, defined as a lamellar count <60, was associated with a risk of AD 15.97 times higher than in normal controls.

The DiGeorge or velocardial facial syndrome, due to a 22q12 deletion, is commonly associated with CTD, and 22q11.2 mutation has been found to be associated with larger aortic root size in TOF (33). Patients with 22q11.2 mutation are more likely to have right aortic arch and pulmonary atresia than non-syndromic patients, so it is unclear, in TOF, if the larger aortic size is due directly to the genetic mutation or rather due to a change in hemodynamics. However, one paper noted that the 22q11.2 deletion itself, even in the absence of CTD, appears to be associated with AD, where AD was noted to have an incidence of 10.8% (34).

Linkage analysis has been used to find novel gene mutations that appear to correlate with TOF and other CTD, but, to date, no studies have been performed to evaluate for novel mutations that explicitly explain AD in CTD.

It is possible that some of the patients in the literature who had CTD and aortic dissection may have had undiagnosed Marfan syndrome or other known connective tissue disease, as the lack of diagnosis may have been retrospectively made on phenotypic, rather than genotypic, grounds. There are numerous variables, including the underlying tissue strength, and varying changes in physiology that frustrate the ability to tease out.

## Future Directions

Further research into AD in CTD will be much more likely to proceed if more patients are found to suffer aortic complications. Next generation sequencing, such as whole exome sequencing, may be very helpful at identifying novel gene mutations that could be responsible for AD in CTD. Genome-wide linkage analysis and exome sequencing together, recently, led to the discovery of a novel *TGFB3* mutation as a cause of syndromic aortic aneurysm and aortic dissection in series of 470 index cases with thoracic aortic aneurysms who had been screened for all known gene mutations associated with thoracic aortic aneurysms (35). Current standard genetic panels to test for aortopathy genes only include 20–25 gene mutations, but these panels will expand greatly as new candidate gene mutations are discovered. *In silico* analysis and more advanced informatics technology will greatly facilitate the ability to translate these findings to clinical practice.

## Summary

The American Heart Association (AHA) and American College of Cardiology (ACC) provide guidelines on the management of thoracic aortic diseases (7), but the most recent guidelines do not provide a clear management decision for how to manage AD in CTD patients. It is exceedingly rare for an aortic dissection to occur in childhood, other than in infancy, due to very severe genetic problems or iatrogenic causes; thus, aortic dissection is largely considered an adult-onset problem. The reader is directed to the current adult congenital heart disease clinical management guidelines (36), which are due for an update in the near future.

The decision of when to intervene for AD in CTD must be weighed on a number of factors, including the number of prior cardiac interventions (and thus, likelihood of morbidity of an elective procedure), the rate of growth of the aorta over time, other lesions that require operative management, and perhaps most importantly, a genetic risk profile. For patients with a strong family history of thoracic aortic aneurysm, aortic dissection, or a known genetic mutation likely to lead to aortic dissection, perhaps, a lower threshold for intervention should be used.

Over time, we may discover advantages to elective aortic root replacement to improve LV–aorta coupling, even in patients not thought to be at acute risk of aortic dissection, as surgical techniques improve. Perhaps, newer discoveries will lead to new therapies that prevent, or even reverse, aortopathies.

Ultimately, only time will tell what the true risk for aortic complications in CTD is, and if the incidence grows over time as this population ages, more research will help determine who is at most risk. It is difficult to determine a risk profile when so few patients have had aortic complications. Ideally, in this era of personalized medicine and high throughput genetic sequencing, every patient will have a unique genetic signature that can be used to tailor his or her unique risk.

## Author Contributions

WK wrote this entire article himself, including reviewing appropriate background literature.

## Conflict of Interest Statement

The author declares that the research was conducted in the absence of any commercial or financial relationships that could be construed as a potential conflict of interest.

## Abbreviations

AD, aortic dilation; CTD, conotruncal defects; DORV, double outlet right ventricle; FBN1, fibrillin-1 gene; TGA, transposition of the great arteries; TOF, tetralogy of Fallot.
